# Using an Overlapping Time Interval Strategy to Study Diagnostic Instability in Mild Cognitive Impairment Subtypes

**DOI:** 10.3390/brainsci9090242

**Published:** 2019-09-19

**Authors:** David Facal, Joan Guàrdia-Olmos, Arturo X. Pereiro, Cristina Lojo-Seoane, Maribel Peró, Onésimo Juncos-Rabadán

**Affiliations:** 1 Department of Developmental Psychology, University de Santiago de Compostela, 15782 Santiago de Compostela, Galicia, Spain; arturoxose.pereiro@usc.es (A.X.P.); cristina.lojo@usc.es (C.L.-S.); onesimo.juncos@usc.es (O.J.-R.); 2 Department of Methodology of Behavioural Sciences, University of Barcelona, 08035 Barcelona, Catalunya, Spain; jguardia@ub.edu (J.G.-O.); mpero@ub.edu (M.P.)

**Keywords:** cognitive aging, mild cognitive impairment, subjective cognitive complaints, conversion to dementia, Bayesian odds ratios, time overlapping intervals, screening and diagnosis

## Abstract

(1) Background: Mild cognitive impairment (MCI) is a diagnostic label in which stability is typically low. The aim of this study was to examine temporal changes in the diagnosis of MCI subtypes by using an overlapping-time strategy; (2) Methods: The study included 435 participants aged over 50 years with subjective cognitive complaints and who completed at least one follow-up evaluation. The probability of transition was estimated using Bayesian odds ratios; (3) Results: Within the different time intervals, the controls with subjective cognitive complaints represented the largest proportion of participants, followed by sda-MCI at baseline and in the first five intervals of the follow-up, but not in the last eight intervals. The odds ratios indicated higher odds of conversion to dementia in sda-MCI and mda-MCI groups relative to na-MCI (e.g., interval 9–15 months—sda-MCI OR = 9 and mda-MCI OR = 3.36; interval 27–33—sda-MCI OR = 16 and mda-MCI = 5.06; interval 42–48—sda-MCI OR = 8.16 and mda-MCI = 3.45; interval 45–51—sda-MCI OR = 3.31 and mda-MCI = 1); (4) Conclusions: Notable patterns of instability consistent with the current literature were observed. The limitations of a prospective approach in the study of MCI transitions are discussed.

## 1. Introduction

Mild cognitive impairment (MCI) is the diagnostic entity used to describe a condition in middle-aged and old adults who experience cognitive decline but without impaired daily functioning [1]. MCI has been indicated as a focal concept for understanding pre-dementia stages in relation to cognitive ageing [2]. Despite some controversy, the following are generally accepted as the core MCI criteria: (i) Evidence of subjective cognitive complaints from patients or their close contacts; (ii) evidence of cognitive impairment in one or more cognitive domains that is greater than expected for the patient’s age and educational background; (iii) preservation or minimal impairment of instrumental activities of daily living; and (iv) non-fulfillment of diagnostic criteria for dementia [3,4,5,6,7]. This consensus also extends to the different subtypes of MCI (amnestic and non-amnestic, single and multi-domain) proposed by Petersen and colleagues [3,6]. Apart from its importance in research on cognitive impairment, MCI is also a multifaceted clinical label, characterized by complex cognitive changes and diagnostic instability in MCI subtypes, conversion to dementia and recovery to normal cognitive aging [8,9,10].

Although major advances in research have led to corresponding changes in the diagnostic criteria for MCI, it remains unclear how cognitive deficits increase over time and how these deficits affect progression to dementia in different MCI subtypes [1,11]. According to Gersteneker and Mast [1], the relationship between MCI subtypes and transition and conversion patterns is not as linear as initially proposed. Amnestic MCI is considered to be the MCI subtype most likely to progress to AD. The diagnostic guidelines produced by the National Institute on Aging- Alzheimer’s disease working groups [5] indicate that the ‘MCI, due to AD’, refers to the symptomatic pre-dementia phase of AD and is characterized by impairment in one or more cognitive domains, typically including episodic memory. Different studies have already shown that multi-domain amnestic MCI (mda-MCI) is the most reliable MCI subtype, with a lower chance of reversion and a higher risk of conversion to dementia over time [12,13].

Although MCI is understood to be an unstable stage and the passage of the time is relevant in its cognitive and clinical manifestations, scant attention has been given in the literature to the time between assessments -but see [9,14]. Establishing this interval may have a considerable impact on the findings of MCI research. For example, longitudinal studies have developed non-linear, plateau models of decline that are dependent on the time of measurement [15]. In the current literature on MCI, the date of initial diagnosis and subsequent measurement points are determined as a function of the study start date; this is problematic as the moment at which participants report subjective cognitive complaints (SCCs) is intrinsically variable [6]. To address this problem, Cloutier et al. [11] suggested aligning the moment retrospectively according to the year in which participants received their diagnosis of AD. Facal et al. [9] proposed an alternative, complementary approach involving an overlapping interval strategy that considers different mid-point stages depending on the time between the baseline and follow-up assessment.

The aim of the present study was to test the capacity of an overlapping time interval strategy to address changes in MCI, by including supplementary follow-up evaluations and broader time intervals. The overlapping interval strategy was applied in the current sample of each time interval to explore whether changes (decline, stability, recovery) are temporally stable or vary according to different time intervals, and also to examine the trajectories of cognitive function in different MCI subtypes within different intervals.

## 2. Materials and Methods

### 2.1. Participants

The current research included 435 participants over 50 years old (range 50–88) included in the on-going Compostela aging study and who completed baseline and at least one follow-up evaluation [16]. All participants were referred to us by general practitioners after attending primary care health centers with subjective cognitive complaints, but with no prior diagnosis of dementia, psychiatric or neurological disorders.

At baseline, the sample comprised 40 participants in the mda-MCI group (multi-domain amnestic MCI), 34 participants in the na-MCI group (no amnestic MCI), 68 participants in the sda-MCI, and 293 participants in the control group with subjective cognitive complaints (SCCs). Age, years of education and results of the cognitive assessment at baseline are shown in Table 1. Group differences were calculated using non-parametric tests (Kruskal-Wallis and Mann-Whitney test) given the differences in sample size between the groups. Diagnoses were made following the Petersen criteria [3,4] updated by Albert et al. [5] and were reached by consensus at research team diagnostic meetings.

In the final sample that completed at least one follow up evaluation, 415 participants completed the first follow-up around 1_1/2_ year after baseline (mean = 17.92 months, S.D. = 3.87, range 10–31 months), and 330 participants completed the second follow up assessment around 3 years after baseline (mean = 37.13 months, S.D. = 5.43, range 27–51 months; this includes 20 participants who did not complete the first follow-up assessment, but who did participate in the second follow-up). Reasons for deviation from the reference times include holidays, health issues and work issues.

### 2.2. Procedure

The baseline evaluations were made between 2 January 2008 and 11 November 2012. Participants undertook wide-ranging cognitive and neuropsychological evaluations, including the Spanish versions of the California Verbal Learning Test (CVLT) [17], the Mini-Mental State Examination (MMSE) [18] and the Cambridge Cognitive Examination—Revised (CAMCOG-R), which include subscales in several cognitive domains (memory, orientation, language, attention and calculation, praxis, perception and executive functioning) [19]. Within the follow-up assessments, all participants again underwent the assessment and were re-diagnosed by the same research team, again by consensus at specific meetings.

At all three evaluation times, the cut-off was 1.5 standard deviations (SDs) below age and education norms on the corresponding tests. All participants who displayed normal cognitive performance (scores higher than cut-off scores on cognitive status and cognitive functioning, including memory) were included in the SCCs group, as they all reported subjective memory complaints. For all MCI participants, the general criteria outlined by Albert et al. [5] were applied: (a) Presence of complaints corroborated by an informant; (b) impairment in one or more cognitive functions; (c) independence in daily living with minimum support or help; and (d) absence of dementia according to the NINCDS-ADRDA and DSM-IV standards. For the specific diagnosis of MCI subtypes, the criteria proposed by Petersen and colleagues [3,6] were applied, including (b1) a score of 1.5 SDs below age standards for short-term and long-term recall measures of the Spanish version of the CVLT for amnestic MCI (aMCI). Two different types of aMCI were diagnosed: Sda-MCI in those participants (b2) who performed 1.5 SDs below age norms in the Spanish version of the CVLT, but performed normally in the MMSE and the CAMCOG-R subscales; and mda-MCI in those participants (b3) who performed 1.5 SDs below norms in the MMSE and at least two sub-scores of the CAMCOG-R representing at least two cognitive functions. Finally, na-MCI was diagnosed in those participants (b4) who performed within the normal range in the Spanish version of the CVLT, but 1.5 SDs below average in at least one of the other subscales of the CAMCOG-R. Single- and multi-domain na-MCI were not differentiated, because of the small sample size in the single-domain na-MCI group. Probable AD or other types of dementia were diagnosed according to the NINCDS-ADRDA and DMS-IV criteria. Progression to dementia was confirmed by consultation of the medical history, and the date of neurological diagnosis was recorded.

The study received approval from the Ethics in Clinical Research Committee of the Galician Government and was conducted in accordance with the provisions of the Declaration of Helsinki as revised in Brazil 2013. Written informed consent was obtained from all participants.

### 2.3. Overlapping Intervals Procedure

According to the variability in the time between the baseline and follow-up evaluations, and the potential effect of time in the study of MCI according to its variability, a time-overlapping interval approach was taken [20] considering 13 mid-point time intervals with a range of 6 months across each interval. This approach enabled a wide range of time distributions to be covered between assessments and also consideration of all participants evaluated, irrespective of the time at which the follow-up was carried out and whether they participated in the first follow up assessment (about one year and a half after the baseline assessment) and/or the second follow up (about three years after the baseline). Seven intervals were included within the time-lapse established for the first follow up assessment (9–15, 12–18, 15–21, 18–24, 21–27, 24–30, 27–33), and seven intervals were also included within the time-lapse established for the second follow up assessment (27–33, 30–36, 33–39, 36–42, 39–45, 42–48, 45–51).

According to this overlapping-interval design, and in order to maximize sample sizes and temporal distributions across successive evaluations, the sample size was different for each interval (see Table 2). According to the reference times for the first (18 months) and the second (36 months) follow-up assessments, the sample sizes tended to be larger in the intervals closer to these times and smaller in the intervening intervals. This approach allows overlapping counts between time intervals, avoiding double-counting of participants by considering the participants only once in those intervals in which months between assessments are included. The range around each mid-point interval was six months, so that adjacent intervals differ in mean time between assessments, but overlap in the time range. Therefore, the mid-point of the 9–15 month group was 12, the mid-point for the 12–18 month group was 15, and so on. The results can, thus, be consulted with the maximum temporal continuum (13 time groups) or with the minimum overlap (seven and six time groups, 9–15, 15–21, 21–27, 27–33, 33–39, 39–45, 45–51 months and 12–18, 18–24, 24–30, 30–36, 36–42, 42–48, respectively). Overlapping intervals are used as independent sample estimates.

### 2.4. Statistical Analysis

The probability of transition from one state to another was estimated using Bayesian estimates, calculated as follows:

*P*(*A_i_*|*B*) = *p*(*B*|*A_i_*)*P*(*A_i_*)/*Σ_k=1_P*(*B*|*A_k_*)*P*(*A_k_*),

where P(Ai) represents the a priori probability, P(B|Ai) represents the probability of transition from Ai to B and, finally, P(Ai|B) represents the a-posteriori probability. In the present study, event Ai assumes the state of the diagnosis at a given time (*t*), and B is the state of diagnosis at time (*t* +1). Evidently, transitions from states Ai to Ak and then to the next state B draw all combinations existing between the diagnostic categories at a given moment and the same categories at the next time. Models of these transitions were generated using P(Ai) and P(Ai|B) from estimates of the proportions observed in the original distributions.

In addition, as the demands of the Bayes’ theorem require exhaustiveness, the odds ratio was obtained from the contrasts, as follows:

*ORij* = *P*(*Ai*|*B*)/*P*(*Aj*|*B*),

with the aim of evaluating the most probable transitions of the pair (i,j) as in the previous expression. Thus, apart from the estimated values of the Bayesian probabilities, more applied values of higher clinical value were obtained. Details of all of the calculations, including those made to obtain Odds Ratios in each interval, are available to readers in Supplementary File S1.

All the mathematical procedures were generated by Excel routines and specific programming in MatLab.

## 3. Results

**Figure 1:**
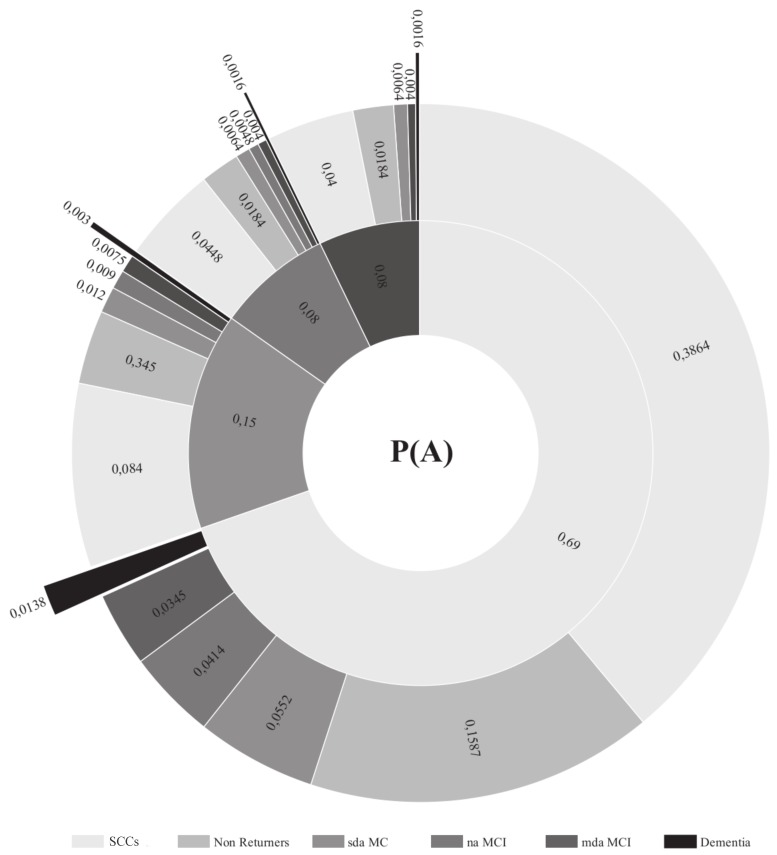
Diagnostic probabilities at baseline (inner circle) and at first follow-up (outer circle). Diagnostic probabilities are presented numerically, as proportions, with the total sum of proportions in the inner and outer circles equaling 1. The naMCI group is absent from the outer circle, but corresponds to the mdaMCI group in the inner circle as no transitions from mdaMCI to naMCI were recorded. SCCs = Subjective cognitive complaints; MCI = mild cognitive impairment; mda = multidomain amnestic; na = non-amnestic; sda = single domain amnestic.

**Figure 2:**
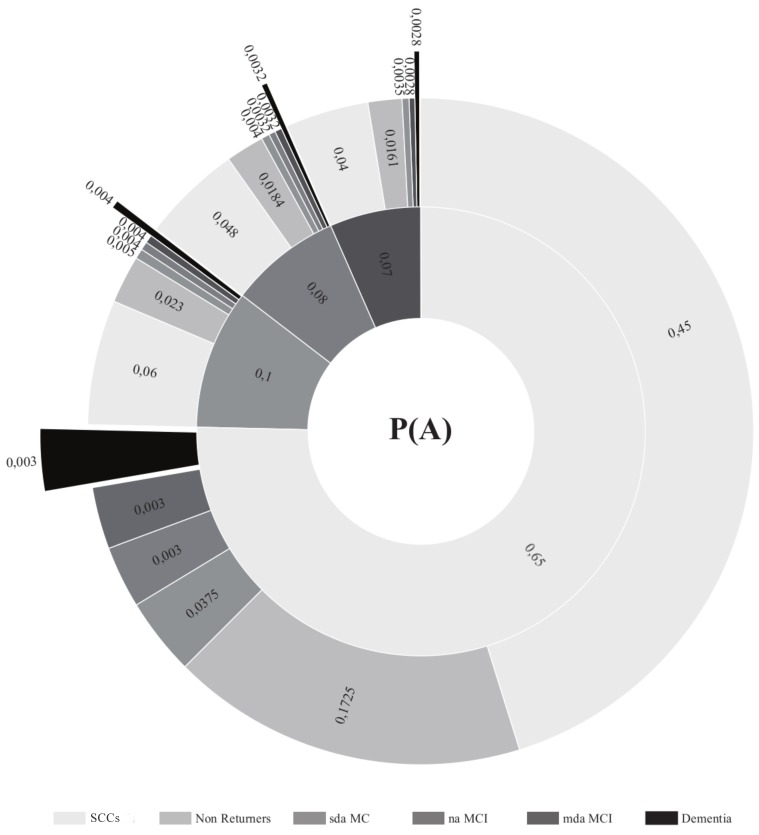
Diagnostic probabilities at first follow-up (inner circle) and at second follow-up (outer circle). Diagnostic probabilities are presented numerically, as proportions, with the total sum of proportions in the inner and outer circles equaling 1. The naMCI group is absent from the outer circle, but corresponds to the mdaMCI group in the inner circle as no transitions from mdaMCI no naMCI were recorded. SCCs = Subjective cognitive complaints; MCI = mild cognitive impairment; mda = multi-domain amnestic; na = non-amnestic; sda = single domain amnestic.

**Figure 3:**
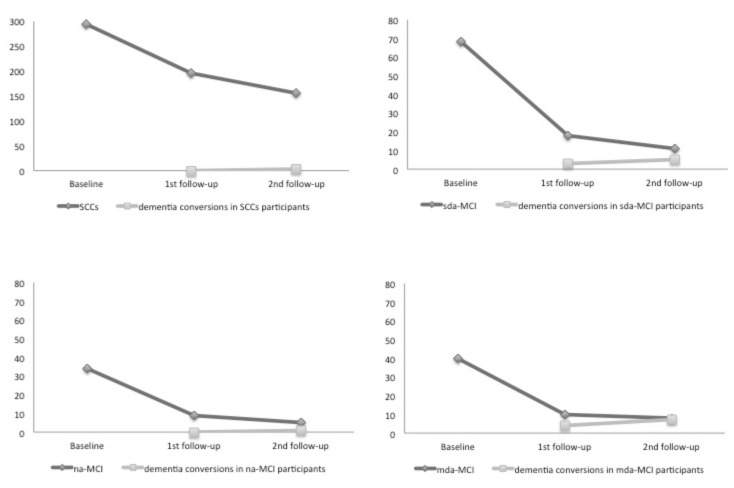
The number of participants whose diagnosis did not change at the follow-up assessments, and the number of participants who converted to dementia. SCCs = Subjective cognitive complaints; MCI = mild cognitive impairment; mda = multi-domain amnestic; na = non-amnestic; sda = single domain amnestic.

The diagnostic probabilities for the different MCI subtypes are presented in Figure 1 and Figure 2. Figure 3 shows those participants whose diagnosis did not change in the follow-up assessments and those participants in the different groups who progressed to dementia. The number of participants in the mda-MCI group who progressed to dementia was 4 at the first follow-up assessment and 7 at the second follow-up assessment; the corresponding numbers in the na-MCI group were 0 at the first follow-up and 1 at the second follow-up assessment; the numbers in the sda-MCI group were 3 at the first follow-up and 5 at the second follow-up; and finally, the corresponding numbers in the SCCs group were 0 at the first follow-up and 4 at the second follow-up. Cases recorded at the first follow-up were also counted at the second follow-up, so that the numbers are cumulative.

**Figure 4:**
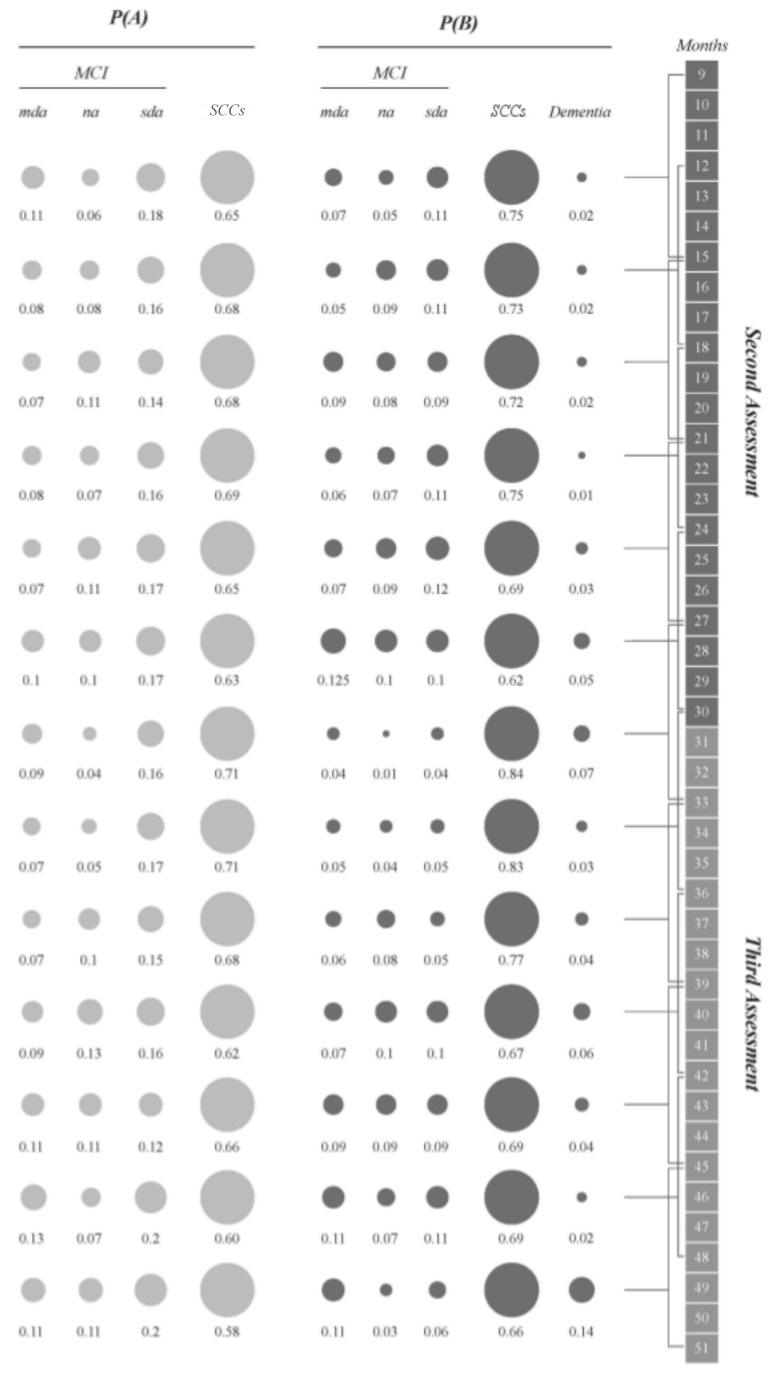
Diagnostic probabilities at baseline **P(A)** and at follow-up **P(B)**. Months between baseline and follow-up assessments are represented on the right-hand side of the diagram. Each line represents one time interval, with brackets in the column on the right showing the overlapping nature of the intervals (e.g., time interval 1 is indicated by the bracket encompassing months 9 to 15 and overlaps with interval 2, indicated by the bracket encompassing months 12 to 18). Diagnostic probabilities are represented at baseline in light grey and at follow-up in dark grey. SCCs = subjective cognitive complaints; MCI = mild cognitive impairment; mda = multidomain amnestic; na = non-amnestic; sda = single domain amnestic.

According to the overlapping time interval strategy, the diagnostic probabilities for the different MCI subtypes at different times are presented in Figure 4. Similar trends were observed for the different time intervals, as follows: (a) SCCs represented the largest proportion of participants, and the proportion tended to increase at follow-up assessments; (b) sda-MCI participants comprised the second largest group at baseline and in the first five intervals of the follow-up, but not in the last eight intervals of the follow-up continuum; (c) the probabilities of diagnosis of mda-MCI and na-MCI were similar at both baseline and follow-up, with slight variations across the time intervals; and (d) there was a small, but increasing, probability of conversion to dementia, represented in the right column of the follow-up section in Figure 3. In this figure, only the cases of conversion to dementia recorded during each time interval are counted.

According to the higher, but temporally variable, probability of sda-MCI at follow-up and to test the theoretical relevance of mda-MCI as the closest point to dementia within the MCI continuum, odds ratios were calculated for each time interval and for sda-MCI and mda-MCI relative to na-MCI. This enabled the probability of conversion to dementia to be determined by comparing the two different amnestic subtypes and considering the non-amnestic subtype as the reference subtype. The conversion odds (Table 3) revealed a higher probability of conversion to dementia in sda-MCI than in na-MCI, which would be expected according to the higher proportion of sda-MCI participants at baseline, and also a higher probability of conversion to dementia in mda-MCI than in na-MCI. 

## 4. Discussion

This study overcomes some limitations of previous research considering diagnostic transition within MCI subtypes and conversion to dementia within different time frames. For example, it includes longer follow-up periods than in previous studies e.g., [9,13] and it uses Bayesian estimates to illustrate the diagnostic transitions more clearly and accurately, representing the reality of daily practice by avoiding an artificial balance of the probability of diagnosis at baseline. In daily gerontological and geriatric practice, longitudinal follow-up and serial cognitive assessments are recommended as they provide a clearer picture of the patient’s baseline and trajectory of cognitive function over time [21], although the optimal timing and cost-effectiveness of longitudinal cognitive assessments remain unclear. In this regard, the transition probabilities calculated using the Bayesian approach with overlapping-interval design are similar to current daily practice in which the time of assessment is variable (i.e., the higher odds in sda-MCI than in mda-MCI compared to na-MCI can be explained by the higher proportion of sda-MCI participants at baseline, in parallel to the higher number of patients with sda-MCI that professionals diagnose in daily practice).

The increase in number of participants classified as SCCs and the decrease in those diagnosed as sda-MCI in the second part of the follow-up assessments indicates the instability of this MCI subtype, as previously highlighted by Malek-Ahmadi in a meta-analysis [22], in which the rate of reversion to normal cognition was approximately 24%. The increase may indicate an excess number of false-positives in diagnosing this particular MCI subtype, which includes patients with impairments only in memory. Nonetheless, the higher odds ratios for conversion to dementia of sda-MCI relative to na-MCI also indicate an important risk of progression of cognitive impairment in the sda-MCI subtype [5,7]. The current clinical criteria, which include a cut-off of <1.5 SD below mean only in memory scores, indicate an unstable subtype, especially when the follow-up assessments are carried out after short intervals. This confirms that other functional, behavioral and biological markers should be included for more accurate diagnosis, as recommended by the American Academy of Neurology (AAN) guidelines on MCI [7]. Follow-up studies are needed because studies that test the actual cognitive status of middle-aged and older adults living in the community with subjective memory complaints appear to be susceptible to false-positive diagnostic errors, mainly regarding single domain MCI subtypes. In this respect, Han et al. [10] recommended considering the diagnostic stability over time and the multiplicity of impaired cognitive domains for managing MCI.

Regarding the rates of conversion to dementia, longitudinal studies have found that non-linear, plateau models of decline (initial decline in memory, followed by a temporary plateau occurring prior to a final rapid decline immediately prior to AD diagnosis) are more accurate than linear models, at least for memory performance [15]. The small, but increasing, probability of conversion to dementia between time intervals observed in our study does not support these findings, although the intervals are relatively short and a slight increase in the case of cognitive decline was detected in the final intervals. However, the rates of conversion to dementia in this study are relatively low [9,23]. Accordingly, adjusting the overlapping time interval strategy included here by including longer intervals and successive follow-up assessments may help to clarify these trends in conversion, e.g., [24].

As already mentioned, this study has some limitations, due to the temporal approach and the rate of variability in the diagnosis of MCI subtypes. Regarding the first limitation, this study used a prospective approach to the complex phenomena of MCI transition and conversion to dementia, adopting overlapping intervals to study the role of time in the MCI diagnostic process. Although the prospective approach has traditionally been used in this field, retrospective approaches involving analysis of data from the moment when patients are diagnosed with Alzheimer’s disease or other types of dementia are increasingly common [6]. For the diagnosis of MCI subtypes, Klekociuk and Summers [13] indicate that it may be necessary to wait until single-domain variants transition to multiple-domain variants in order to identify genuine cases, particularly when recruiting prospective samples with subjective memory complaints—as in the present study. Regarding the variability in diagnosis of MCI subtypes, in this study, we measured verbal learning and memory with the Spanish version of the CVLT [9] and other cognitive functions with the CAMCOG-R. The use of other specific cognitive measures of memory and non-memory functions [25,26], as well as functional, behavioral and biological measures, could minimize the diagnostic instability [7]. It has been suggested that the number of false-positive can also be reduced by using empirical statistical approaches to identify MCI, such as cluster analysis based on the actual cognitive performance of the participants rather than previously established cut-off scores [27,28,29]. In this regard, Edmonds et al. [27] suggest that the inclusion of self-reported cognitive complaints as a core MCI diagnostic criterion may yield higher false-positive rates, as amnestic MCI participants tend to underestimate the level of cognitive impairment.

## 5. Conclusions

In summary, use of the time-overlapping intervals strategy enabled us to study the diagnostic instability in MCI subtypes, contributing further knowledge about temporal effects and the time between follow-up assessment on MCI trajectories, and revealing distinctive trajectories of diagnostic changes, especially in the sda-MCI subtype. Nevertheless, the classical prospective approach has some limitations in relation to studying diagnostic instability in MCI. These limitations must be addressed in future studies by using a more comprehensive neuropsychological and biological approaches, longer time intervals between successive follow-ups, and combining prospective and retrospective diagnostic approaches.

## Figures and Tables

**Table 1:** Mean values and standard deviations (in parentheses) of the demographic and cognitive measures in each group at baseline.

	mda-MCI	na-MCI	sda-MCI	SCCs	Group Differences χ² (gl)	Group Comparisons
Age	72.42 (8.33)	67.12 (8.82)	69.29 (9.36)	65.49 (9.05)	24.83 **	mda-MCI > naMCI, SCCs; sda-MCI>SCCs
Years of education	9.68 (8.82)	8.29 (3.75)	9.40 (4.17)	9.92 (4.70)	1.81	
Memory complaints—participant	19.43 (4.69)	20.35 (3.45)	19.03 (4.69)	18.81 (4.51)	8.53 *	naMCI > SCCs
Memory complaints—proxy	17.97 (4.54)	18.03 (4.76)	16.84 (4.46)	15.49 (4.23)	17.03 **	mda-MCI, naMCI > sdaMCI, SCCs; sda-MCI > SCCs
CVLT Short Delay Free Recall	3.10 (2.03)	9.18 (2.21)	3.88 (2.05)	10.34 (2.70)	228.58 **	SCCs, na-MCI > mda-MCI, sda-MCI; sda-MCI > mda-MCI
CVLT Long Delay Free Recall	3.82 (3.19)	9.76 (2.69)	5.12 (3.06)	11.14 (2.83)	184.80 **	SCCs, sda-MCI, na-MCI > mdaMCI; SCCs> na-MCI, sda-MCI; na-MCI>sda-MCI
CAMCOG-R memory	15.50 (3.80)	18.56 (2.87)	18.53 (3.88)	21.26 (2.78)	99.18 **	SCCs, sda-MCI, na-MCI > mdaMCI; SCCs > na-MCI, sda-MCI
CAMCOG-R orientation	7.98 (1.46)	9.29 (0.80)	9.31 (0.83)	9.64 (0.63)	79.19 **	SCCs, sda-MCI, na-MCI > mdaMCI; SCCs > na-MCI, sda-MCI
CAMCOG-R language	22.77 (2.36)	23.53 (2.00)	24.90 (2.43)	25.60 (2.40)	54.55 **	SCCs, sda-MCI > mdaMCI, na-MCI
CAMCOG-R attention - calculation	5.03 (2.21)	4.29 (1.73)	7.25 (1.70)	7.05 (1.97)	100.90 **	SCCs, sda-MCI > mdaMCI, na-MCI
CAMCOG-R praxis	9.10 (2.53)	10.03 (1.71)	10.71 (1.47)	11.14 (1.19)	48.01 **	SCCs, sda-MCI > mdaMCI, na-MCI
CAMCOG-R perception	5.97 (1.64)	6.50 (1.28)	6.53 (1.48)	7.06 (1.44)	21.68 **	SCCs, sda-MCI > mdaMCI; SCCs > na-MCI, sda-MCI
CAMCOG-R executive function	13.22 (3.94)	15.35 (4.20)	15.93 (4.28)	18.42 (4.20)	58.84 **	SCCs, sda-MCI > mdaMCI; SCCs > na-MCI, sda-MCI

** *p* < 0.01 * *p* < 0.05. Note: CVLT = california verbal learning test; CAMCOG-R = cambridge cognitive assessment-revised; mda-MCI = multi-domain amnestic Mild Cognitive Impairment; na-MCI = non-amnestic mild cognitive impairment; sda-MCI = single-domain amnestic mild cognitive impairment; SCCs = subjective cognitive complaints.

**Table 2:** Sample size in each overlapped time interval.

Interval	mda-MCI	na-MCI	sda-MCI	SCCs
9–15 months	6	3	10	35
12–18 months	14	15	30	125
15–21 months	16	16	31	155
18–24 months	13	11	22	105
21–27 months	5	8	13	49
24–30 months	4	4	7	25
27–33 months	6	3	11	47
30–36 months	9	7	21	98
33–39 months	10	15	21	98
36–42 months	8	12	14	56
39–45 months	8	8	9	51
42–48 months	6	3	9	27
45–51 months	4	4	9	18

**Table 3:** Odds ratios for conversion to dementia in sda-MCI and mda-MCI groups relative to na-MCI.

Interval	sda-MCI OR (95% CI)	mda-MCI OR (95% CI)
9–15 months	9 (8.20–9.80)	3.36 (3.06–3.66)
12–18 months	4 (3.35–4.65)	1 (1.00–2.12)
15–21 months	1.62 (1.22–2.02)	0.41 (0–1.21)
18–24 months	5.22 (4.67–5.77)	1.31 (1.08–1.34)
21–27 months	2.39 (1.45–3.34)	0.41 (0–1.21)
24–30 months	2.89 (2.10–3.68)	1 (1.00–2.12)
27–33 months	16 (12.10–19.90)	5.06 (4.85–5.27)
30–36 months	11.56 (10.10–12.45)	1.96 (1.52–2.40)
33–39 months	2.25 (1,10–3.40)	0.49 (0–1.21)
36–42 months	1.51 (1.10–1.92)	0.48 (0–1.21)
39–45 months	1.19 (1.00–1.77)	1 (1.00–2.12)
42–48 months	8.16 (6.80–9.54)	3.45 (2.90–4.05)
45–51 months	3.31 (2.80–3.82)	1 (1.00–2.12)

## Supplementary Materials

The following are available online at https://www.mdpi.com/2076-3425/9/9/242/s1, File S1: The calculations made to study transitions in each time interval.
